# Electrochemical Sensing Platform Based on Metal Nanoparticles for Epinephrine and Serotonin

**DOI:** 10.3390/bios13080781

**Published:** 2023-08-01

**Authors:** Sorina Alexandra Leau, Cecilia Lete, Cristian Matei, Stelian Lupu

**Affiliations:** 1 Department of Electrochemistry and Corrosion, Institute of Physical Chemistry “Ilie Murgulescu” of the Romanian Academy, 202 Splaiul Independentei, 060021 Bucharest, Romania; 2 Department of Analytical Chemistry and Environmental Engineering, Faculty of Chemical Engineering and Biotechnologies, University “Politehnica” of Bucharest, 1-7 Polizu Gheorghe, 060042 Bucharest, Romania; 3 Department of Inorganic, Physical Chemistry and Electrochemistry, Faculty of Chemical Engineering and Biotechnologies, University “Politehnica” of Bucharest, 1-7 Polizu Gheorghe, 060042 Bucharest, Romania

**Keywords:** nanocomposite materials, metal nanoparticles, sinusoidal currents, serotonin, epinephrine

## Abstract

A sensing platform based on nanocomposite materials composed of gold metal nanoparticles (AuNPs) and conducting polymer (CP) matrix has been developed for serotonin and epinephrine detection. The CP-AuNPs nanocomposite materials have been synthesized onto glassy carbon electrodes (GCE) by using novel electrochemical procedures based on sinusoidal currents (SC). The SC procedures ensured good control of the metal nanoparticles distribution, increased electrochemical surface area, and enhanced analytical performance. The proposed sensing platform displayed good analytical performance toward serotonin and epinephrine detection. A wide linear analytical response toward epinephrine in the range from 10 to 640 μM and a low detection limit of 1.4 μM epinephrine has been obtained. The sensing platform has also displayed a linear response toward serotonin in the range from 10 to 320 μM, with a detection limit of 5.7 μM serotonin. The sensing platform has been successfully applied in the analysis of epinephrine and serotonin in real samples of tap water and urine with good accuracy.

## 1. Introduction

The monitoring of neurotransmitters like dopamine, serotonin, and epinephrine represents a very active research topic in the management of chronic diseases like Alzheimer’s and Parkinson’s. These neurotransmitters play key roles in the functioning of the central nervous system and their abnormal levels are associated with neurological diseases. Consequently, the quantification of the neurotransmitters by various analytical methodologies including optical methods, chromatography, and capillary electrophoresis with low detection limits have been applied [1,2,3,4]. These analytical techniques provide high sensitivity, selectivity, and precise quantification of various neurotransmitters at low concentration levels in real samples with complex compositions. However, these methodologies require expensive and sophisticated experimental setups. As an alternative to these approaches, fast, sensitive, reliable, and cost-effective electrochemical-based techniques have been investigated during the last few years [5,6]. The use of sensing materials based on nanostructured conducting polymers and inorganic metal nanoparticles demonstrates improved analytical performance toward the detection of various neurotransmitters such as serotonin, dopamine, and epinephrine [7]. The synthesis of nanostructured composite materials composed of organic conducting polymers and metal nanoparticles could be achieved by chemical and/or electrochemical routes. The electrochemical route ensures better control of the thickness of the electrodeposited sensing materials and provides a fast and versatile approach for metal nanoparticle synthesis. The conducting polymer matrix provides a favorable microenvironment for the in situ preparation of metallic nanoparticles (MeNPs) such as Pt, Au, and Ag, to name but a few. In addition, the organic polymeric matrix improves the selectivity of the analytical measurements, while the metallic nanoparticles increase the sensitivity thanks to their catalytic activity. This approach has demonstrated several benefits like high sensitivity, fast analysis time, reliability, and selectivity in the electrochemical sensing of various neurotransmitters [8,9,10,11,12]. In a recent study, a serotonin-sensitive electrochemical sensor based on polypyrrole (PPy) and AuNPs has been developed [13]. The sensing composite material has been synthesized in two steps. Firstly, the electropolymerization of the pyrrole monomer onto graphite-based screen-printed electrode was achieved via multipulse amperometry. Afterward, the AuNPs were electrogenerated by a potentiodynamic method in an aqueous solution containing the metallic precursor. The PPy-AuNPs composite materials displayed increased sensitivity and a low detection limit. The improved overall analytical performance was due to the increase in the electroactive surface area provided by the PPy matrix and the catalytic effects brought by AuNPs. The proposed electrochemical sensor was successfully employed in the quantification of serotonin in serum samples. In another study, an electrochemical sensor based on the conducting polymer polyaniline (PANI) and Au nanoparticles (AuNPs) for dopamine (DA) detection has been developed [14]. The electrodeposition of AuNPs has been achieved by potential cycling onto the PANI layer. The electrochemical sensor has been successfully applied in dopamine determination with a linear range of 1–100 μM and a detection limit of 0.86 μM DA.

The main electrochemical methods used in the preparation of conducting polymer–metal nanoparticles composite sensing materials are the potentiostatic, galvanostatic, and potentiodynamic ones. In addition to these methods, current pulse-based methods have been also employed in the development of electrochemical sensors. Compared to chemical preparation procedures for metal nanoparticle-based composite materials [15,16], the electrochemical ones provide better control of the thickness of the polymeric matrix and the size of the metal nanoparticles [17,18,19]. Recently, we have developed novel preparation procedures based on the use of sinusoidal voltages for the electrodeposition of conducting polymers and metal nanoparticles composite materials [20,21,22,23]. These novel procedures ensure the preparation of conducting polymeric coatings characterized by increased roughness that has a beneficial effect on improving the sensitivity of the analytical measurements. The main advantages of the electrochemical methods used in the elaboration of various sensors for neurotransmitter detection such as sensitivity, cost-effective methodology and tools, and reliable and fast analytical measurements are underpinning the research in this field. However, there is also a keen interest in solving some drawbacks and issues like low reproducibility in the preparation of sensing materials from one batch to another, the fouling of the electrode surface with chemical reaction products or by-products, and the reduced stability of the developed electrochemical sensors over longer periods of time. Therefore, the development of various procedures for the preparation of sensing composite materials with better sensitivity, selectivity, and reliability is pursued by many research directions. For instance, the design of novel electrochemical preparation procedures aims to overcome the low stability of the sensors by ensuring the fast production of several sensors in a reasonable time. Moreover, the electrochemical preparation procedures could be easily applied to various electrode substrates like metals, semiconductors, or screen-printed electrodes for the development of disposable sensors.

In the present study, a novel preparation method based on sinusoidal currents (SC) of fixed frequency and amplitude has been employed in the development of an electrochemical sensing platform for serotonin and epinephrine determination. The poly(3,4-ethylenedioxythiophene) (PEDOT)–AuNPs composite material was electrodeposited onto glassy carbon electrodes by means of an SC procedure where a sinusoidal current was superimposed on a constant current. The obtained modified electrodes have been characterized by a range of techniques including electrochemical methods and scanning electron microscopy. The proposed electrochemical sensors have been applied in the analytical determination of serotonin and epinephrine.

## 2. Materials and Methods

### 2.1. Materials and Apparatus

All the chemicals were of analytical grade and used as received. 3,4-ethylenedioxythiophene (EDOT, 99%), 5-hydroxytryptamine hydrochloride, (±)-epinephrine hydrochloride, sodium tetrachloroaurate(III) dihydrate (NaAuCl_4_·2H_2_O), lithium perchlorate (LiClO_4_), potassium nitrate (KNO_3_), monobasic potassium phosphate (KH_2_PO_4_), dibasic potassium phosphate (K_2_HPO_4_), sulfuric acid (H_2_SO_4_), potassium hexacyanoferrate (III) (K_3_Fe(CN)_6_), and sodium hexacyanoferrate (II) (Na_4_Fe(CN)_6_·10H_2_O) were purchased from Sigma-Aldrich. All aqueous solutions were prepared using double distilled water. Stock solutions were prepared daily and stored in the refrigerator.

The electrochemical experiments were performed using a potentiostat–galvanostat Autolab 302N (EcoChemie, Utrecht, The Netherlands), connected to a PC via USB interface and controlled with the software NOVA 2.1.5 (Metrohm Autolab B.V., Utrecht, The Netherlands). The electrochemical measurements were performed in a cell (Metrohm) containing three electrodes: the working electrode was a glassy carbon disk electrode with diameter of 3 mm (Metrohm), a glassy carbon rod (Metrohm) was used as counter electrode, and an Ag/AgCl/KCl (3M) (Metrohm) electrode served as reference electrode. The potential values are expressed versus the Ag/AgCl/KCl (3M) reference electrode. All measurements were performed at room temperature. Prior to the measurements, all the aqueous solutions were bubbled with Ar 99.999% and an Ar blanket was maintained over the solutions during the measurements.

The SC method was implemented with the FRA module of the potentiostat. The electrochemical impedance spectra were recorded in the frequency range from 10 kHz to 0.05 Hz, using an excitation signal with amplitude of 5 mV (rms), at the open circuit potential, in an aqueous solution containing 5 mM [Fe(CN)_6_]^3−/4−^ and 0.5 M KNO_3_.

The morphology of PEDOT-AuNPs composite materials has been investigated by means of scanning electron microscopy (SEM) using a Tescan Vega3 LMH instrument equipped with a BSE detector and EDS spectrometer.

### 2.2. Synthesis Methods for CP-AuNPs Nanocomposite Material

**Scheme 1:**
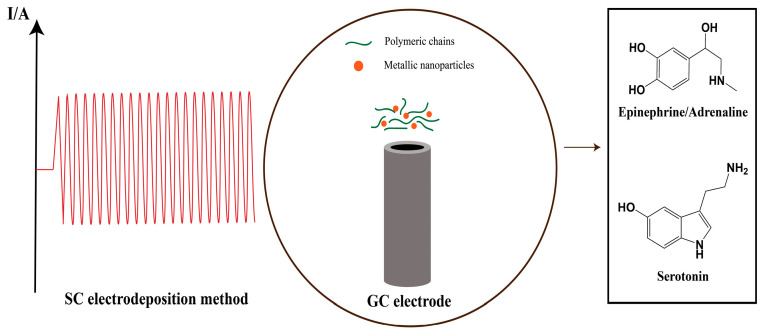
Construction of the PEDOT-AuNPs-based sensing platform.

The synthesis of the CP-AuNPs nanocomposite material has been achieved by means of an innovative preparation procedure based on the use of sinusoidal currents. The SC procedure consists of the application of a sinusoidal current of selected frequency and amplitude over a constant current. The following experimental parameters of the SC procedure have been optimized: the amplitude of the sinusoidal current, *I_sin_*, the frequency of the sinusoidal current, *f*, the value of the constant current *I_dc_*, and the electrodeposition time, *t_dep_*. The CP-AuNPs nanocomposite materials were prepared in two steps by SC procedure: (i) In the first step, the electrodeposition of the PEDOT layer has been performed from an aqueous solution containing 10 mM EDOT, and 0.1 M LiClO_4_, using the following optimized SC parameters: *I_dc_* = 10 μA, *I_sin_* = 25 μA, *f* = 100 mHz, *t_dep_* = 300 s. (ii) In the second step, the Au metal nanoparticles have been electrodeposited in-situ onto the PEDOT layer from an aqueous solution containing 5 mM NaAuCl_4_ and 0.5 M H_2_SO_4_ using the following optimized experimental SC parameters: *I_dc_* = −20 μA, *I_sin_* = 20 μA, *f* = 50 mHz, *t_dep_* = 100 s. These electrochemical parameters of the SC procedure have been selected after the optimization of the following experimental parameters: (i) PEDOT deposition, *I_dc_* of 10 and 25 μA, *I_sin_* of 20 and 25 μA, frequencies of 50 and 100 mHz, deposition times of 300 and 600 s, have been studied, respectively; (ii) AuNPs electrodeposition, *I_dc_* of −1, −20, and −70 μA, *I_sin_* of 0.35, 20, and 35 μA, frequencies of 50 and 100 mHz, deposition times of 100 and 200 s, have been investigated, respectively. A schematic representation of the construction of the PEDOT-AuNPs-based sensing platform is depicted in Scheme 1.

### 2.3. Analytical Applications of CP-AuNPs Nanocomposite Material

The electrochemical sensor based on CP-AuNPs composite material has been applied in the detection of epinephrine and serotonin. The electrochemical detection was performed by using cyclic voltammetry technique in phosphate buffer aqueous solution of pH 7. The analytical calibration curves for epinephrine and serotonin have been constructed by multiple standard addition protocols by adding standard aliquots in the phosphate buffer aqueous solution of pH 7 using the GCE-PEDOT-AuNPs-based electrochemical sensor. A known volume of 10 mL phosphate buffer solution of pH 7 was introduced in the electrochemical cell. The tested concentrations of epinephrine and serotonin were as follows: 10, 20, 40, 80, 160, 320, and 640 μM. The analytical performance of the developed sensor in terms of sensitivity, linear response range, detection and quantification limits, repeatability, and reproducibility has been evaluated. The practical applicability of the developed sensors has been investigated by epinephrine and serotonin detection in real samples. The tap water was collected from the tap water facility in the laboratory. The urine sample was collected from a healthy volunteer with informed consent. The sample of tap water has been diluted at a ratio of 1:2.5 with 0.1 M phosphate buffer solution prior to the analysis. In the case of the urine sample, the analysis was performed after the dilution of the sample at a ratio of 1:10 with 0.1 M phosphate buffer solution. The standard addition protocol has been applied for real samples of tap water and urine analysis.

## 3. Results and Discussion

### 3.1. Characterization and Optimization of PEDOT-AuNPs Sensing Material

**Figure 1:**
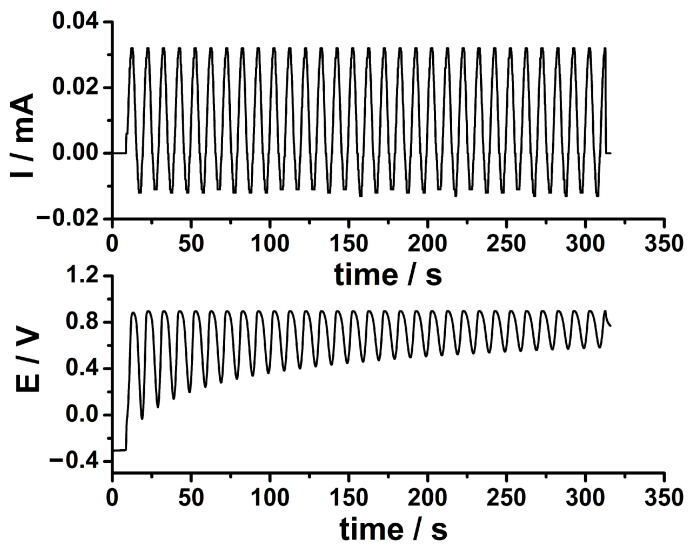
The sinusoidal current applied at the GCE during the electrodeposition of PEDOT coating by SC procedure from an aqueous solution containing 10 mM EDOT and 0.1 M LiClO4. Experimental parameters: *I_dc_* = 10 μA, *I_sin_* = 25 μA, *f* = 100 mHz, *t_dep_* = 300 s (**upper panel**). The potential response of the system after the application of the sinusoidal current (**lower panel**).

The PEDOT-AuNPs sensing material was prepared onto GC electrode according to the preparation procedure based on SC and the fabrication process was monitored by cyclic voltammetric measurements in the presence of a soluble redox probe, that is [Fe(CN)_6_]^4−^. In the first step, the electropolymerization of the 3,4-ethyelendioxythiophene monomer onto GCE by using the SC procedure has been performed. The applied sinusoidal current and the resulting potential response recorded during the electropolymerization process are depicted in Figure 1.

The applied sinusoidal current (see Figure 1) ensured the electrochemical polymerization of the EDOT monomer due to the resulting potential with a sinusoidal shape in the range of ca. 0.30 to 0.90 V. It is well known that the EDOT electropolymerization takes place in a potential range comprised between 0.80 and 0.95 V. The potential response of the system depicted in Figure 1 is comprised of this potential range attesting to the capability of the SC preparation procedure to perform the PEDOT electrodeposition. It is worth noting that only the anodic part of the applied sinusoidal current with respect to the constant current value is actually ensuring the electropolymerization of the EDOT monomer. The amount of PEDOT, *W_PEDOT_*_,_ could be estimated by using this equation [23]: *W_PEDOT_ = Q/nFA*, where *Q* stands for the electrical charge used in the electrodeposition of PEDOT by SC procedure, (48.9 mC cm^−2^), *n* is the number of electrons transferred per EDOT monomer molecule (*n* = 2.25) [24], *F* represents the Faraday constant (96,485 C mol^−1^), and *A* is the geometric surface area (0.071 cm^2^). An amount of 2.3 × 10^−7^ mol cm^−2^ PEDOT could be obtained in this way assuming an efficiency of 100% of the electrochemical polymerization process. These optimized experimental parameters demonstrate the usefulness of the SC method in the preparation of the PEDOT coating.

**Figure 2:**
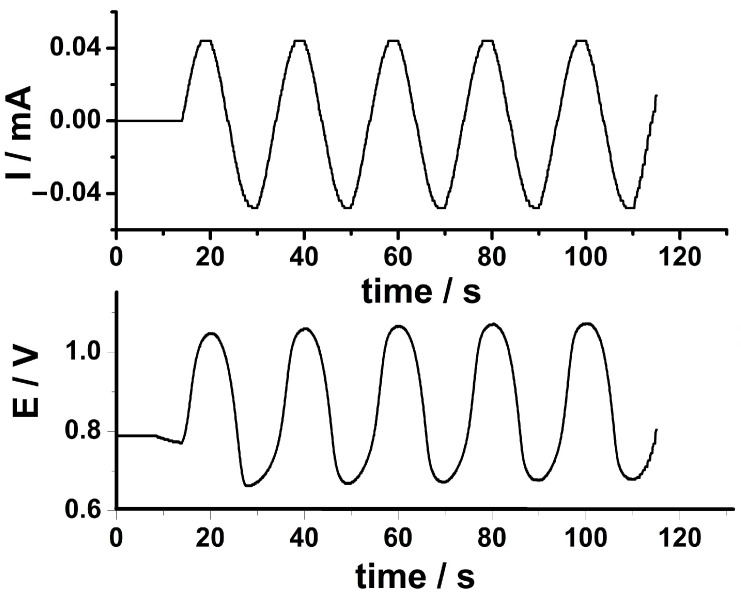
The sinusoidal current applied during the electrodeposition of AuNPs onto GCE/PEDOT electrode by SC procedure from an aqueous solution containing 5 mM NaAuCl_4_ and 0.5 M H_2_SO_4_. Experimental parameters: *I_dc_* = −20 μA, *I_sin_* = 20 μA, *f* = 50 mHz, *t_dep_* = 100 s (**upper panel**). The potential response of the system after the application of the sinusoidal current (**lower panel**).

After the electrodeposition of the PEDOT coating onto GCE, the in situ electrodeposition of AuNPs has been achieved from an aqueous solution containing the metallic precursor using the SC procedure. The applied SC signal and the resulting potential recorded during the AuNPs electrodeposition are depicted in Figure 2.

The experimental parameters for AuNPs electrodeposition have been optimized in terms of constant current, amplitude of the sinusoidal current, frequency of the sinusoidal current, and deposition time. A sinusoidal current of 20 μA amplitude and frequency of 50 mHz has been superimposed on a cathodic constant current of (−20) μA in order to achieve the in situ electrodeposition of AuNPs onto the GCE-PEDOT modified electrode. The potential response of the system varied between 0.65 and 1.03 V and displayed also a sinusoidal shape similar to the applied SC signal. The electrodeposition of AuNPs takes place during the cathodic part of the applied SC signal below the potential value of 0.80 V. In this way, the contribution of the SC signal is well separated from the contribution of the constant current attesting the feasibility of the sinusoidal current procedure in the preparation of the PEDOT-AuNPs composite material. The amount of AuNPs (*W_AuNPs_*) could be estimated using the following equation [23]: *W* = (*Q_AuNPs_M*)/(*nF*), where *Q_AuNPs_* represents the electrical charge used in the electrodeposition of AuNPs using the SC procedure (19.7 mC cm^−2^), *M* stands for the atomic weight of Au (196.96 g mol^−1^), *n* is the number of electrons transferred in the electrodeposition process (*n* = 3) and *F* is the Faraday constant (96,485 C mol^−1^). An amount of AuNPs of 13.4 µg cm^−2^ has been obtained in this way assuming an efficiency of 100% of the electrodeposition process.

**Figure 3:**
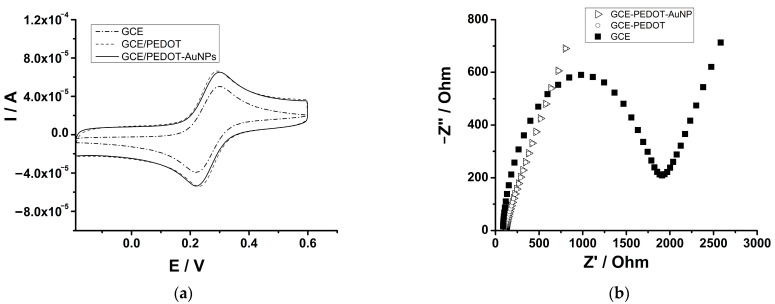
(**a**) CVs recorded at GCE, GCE-PEDOT, and GCE-PEDOT-AuNPs electrodes in aqueous solution containing 5 mM Na_4_Fe(CN)_6_ and 0.5 M KNO_3_. Potential scan rate: 50 mV/s. (**b**) EIS spectra recorded at GCE, GCE-PEDOT, and GCE-PEDOT-AuNPs electrodes in aqueous solution containing 5 mM [Fe(CN)_6_]^3/4−^ and 0.5 M KNO_3_.

The preparation of the GCE-PEDOT-AuNPs-based sensor has been monitored by cyclic voltammetry using a soluble redox probe. A comparison of the GCE-PEDOT-AuNPs sensor with the unmodified electrode GCE and the GCE-PEDOT electrode has been performed. Figure 3a displays the CV traces recorded at GCE, GCE-PEDOT, and GCE-PEDOT-AuNPs electrodes in an aqueous solution containing 5 mM Na_4_Fe(CN)_6_ and 0.5 M KNO_3_. There is an increase in the anodic peak current for GCE-PEDOT (*I_pa_* = 6.6 μA) and GCE-PEDOT-AuNPs (*I_pa_* = 6.5 μA) electrodes compared to the unmodified GC electrode (*I_pa_* = 4.8 μA). This increase in the peak current demonstrates the capability of modifying layers to enhance the electron transfer at the electrode/solution interface. The electrochemical active surface areas (*ECAS*) for GCE-PEDOT and GCE-PEDOT-AuNPs electrodes have been estimated using cyclic voltammetry data. The *ECAS* has been estimated using the Randles–Sevcik equation and the slope of the anodic peak current versus the potential scan rate dependence plot. A diffusion coefficient value of 6.67 × 10^−6^ cm^2^ s^−1^ for ferrocyanide ion has been applied in the calculation of the *ECAS* values. The *ECAS* values of 0.11 cm^2^ and 0.15 cm^2^ for GCE-PEDOT and GCE-PEDOT-AuNPs have been obtained, respectively. The roughness factors have been estimated based on the geometric surface area of the unmodified GC electrode and the obtained values were 3.5 and 3.7 for GCE-PEDOT and GCE-PEDOT-AuNPs electrodes, respectively. The obtained *ECAS* value of the proposed GCE-PEDOT-AuNP modified electrode demonstrates the increased electron transfer capability of the sensing material. The electron transfer capability of the PEDOT-AuNPs composite coating has been also investigated by means of electrochemical impedance spectroscopy (EIS). The EIS measurements were performed over the frequency range from 10 kHz to 0.05 Hz using a small amplitude of the AC perturbation signal of 5 mV at the open circuit potential of the redox couple. The EIS spectra recorded at the GCE, GCE-PEDOT, and GCE-PEDOT-AuNPs electrodes in the presence of a soluble redox couple, that is Na_4_Fe(CN)_6_/K_3_Fe(CN)_6_, are depicted in Figure 3b. In the case of GCE-PEDOT and GCE-PEDOT-AuNPs electrodes, the impedance spectra display the Warburg diffusion element at low frequency with a slope close to 1, while the semicircle at high frequency due to the charge transfer resistance element is not visible because of the fast electron transfer process at the electrode/solution interface. The spectrum of the unmodified GC electrode displayed a large semicircle at the high-frequency range due to the charge transfer process, and a Warburg diffusion element at the low-frequency range. The charge transfer resistance (*Rct*) has been estimated for GCE, GCE-PEDOT, and GCE-PEDOT-AuNPs using a Randles equivalent electrical circuit composed of the usual components: solution resistance (*Rs*), charge transfer resistance (*Rct*), constant phase element (*CPE*), and Warburg impedance (*W*). The following *Rct* values have been obtained: 332.1 Ω, 307.7 mΩ, and 22.9 mΩ for GCE, GCE-PEDOT, and GCE-PEDOT-AuNPs electrodes, respectively. The decreased charge transfer resistance of the PEDOT-AuNPs composite sensing layer demonstrates an increased electron transfer capability for the redox probe, which ensures the improvement of the analytical response of the sensor towards the target analyte.

The in situ electrodeposition of AuNPs has been performed under optimum experimental conditions that ensure the formation of dispersed nanoparticles and avoid the formation of an Au metallic layer on top of PEDOT coating. If the electrodeposition time for AuNPs is increased over 100 s, a decrease in the anodic peak currents in the presence of the redox probe is observed. Therefore, the experimental parameters for AuNPs electrodeposition were optimized in order to obtain a similar electrochemical behavior of the redox probe as in the case of the PEDOT coating. This experimental approach ensures improved electrochemical properties of the modifying material PEDOT-AuNPs by combining the benefits of the PEDOT matrix and the presence of AuNPs.

**Figure 4:**
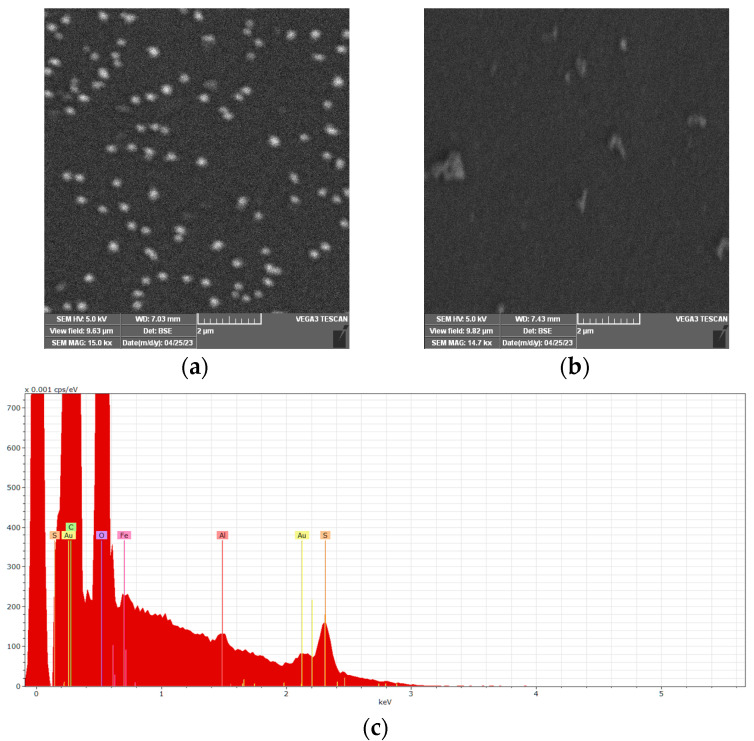
SEM images of PEDOT-AuNPs (**a**), PEDOT (**b**) materials, and (**c**) the EDX spectrum of PEDOT-AuNPs material.

The morphological characterization of PEDOT-AuNPs composite material and PEDOT coating have been performed by using the SEM technique and the obtained data are displayed in Figure 4. The PEDOT coating displayed large grains aggregates dispersed over the surface. The SEM measurements demonstrated the presence of AuNPs in the PEDOT matrix with a mean diameter of 196.6 ± 24.5 nm. A homogeneous distribution of AuNPs over the PEDOT matrix with good control of the nanoparticles’ diameter could be observed. The EDX spectrum of the PEDOT-AuNPs composite material (Figure 4c) displays the signal specific to Au and this demonstrates the successful deposition of AuNPs by using the SC procedure. The signal related to Fe could be due to the measurements performed in ferro/ferricyanide-containing solution during the preparation of the composite material, while the signal of Al could be due to the mechanical polishing of the GC electrode substrate with alumina powder. However, these species do not influence the properties of the composite material. Considering the amounts of AuNPs and PEDOT estimated by electrical charge data, the calculated molar ratio Au:EDOT is 1:3.38, which correlates relatively well with the ratio obtained from the EDX spectral analysis, 1:4.88. The difference can be explained by the fact that the determination of the charge involved the entire surface of the electrode, while the EDX analysis was conducted on a narrow, randomly selected area. These results confirm the successful electrodeposition of AuNPs by means of the SC procedure.

### 3.2. Analytical Applications

#### 3.2.1. Detection of Epinephrine

**Figure 5:**
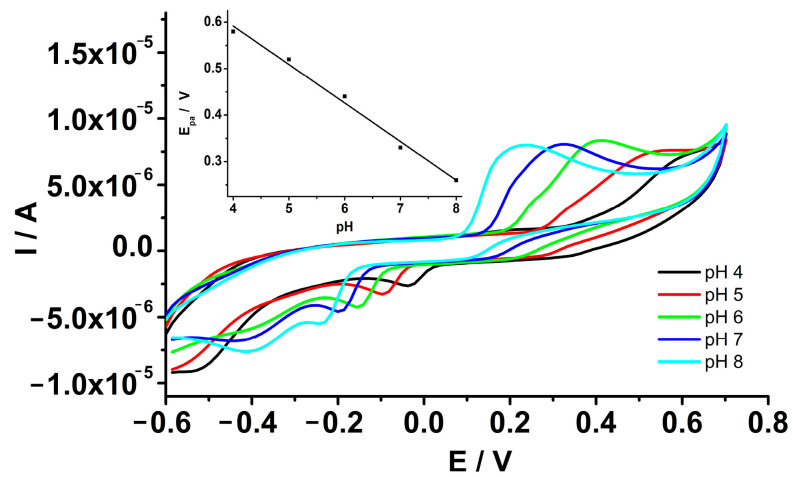
CVs recorded at GCE-PEDOT-AuNPs sensor in aqueous solution containing 0.1 M phosphate buffer and 320 μM epinephrine at pH values ranging from 4 to 8. Potential scan rate: 50 mV/s. Inset: the anodic peak potential versus pH dependence plot.

The electrochemical behavior of epinephrine was investigated at GCE-PEDOT-AuNPs based sensor by means of cyclic voltammetry technique in aqueous buffered solution. In view of the analytical applications of the proposed sensor, the behavior of epinephrine in solutions of various pH values has been studied. The influence of pH on epinephrine oxidation was estimated by cyclic voltammetry in the pH range from 4 to 8. Figure 5 shows the cyclic voltammograms recorded at the GCE-PEDOT-AuNPs sensor in an aqueous solution of various pH values. It is noted that the anodic peak potential *E_pa_* for epinephrine oxidation shifts toward less positive values with the increase in pH. The linear regression equation of the peak potential dependence on pH was as follows: *E_pa_ (V) =* 0.924–0.083 pH, with correlation coefficient *r* = 0.9955. The slope of the *E_pa_* versus pH plot was −0.083 V/pH. The obtained slope is close to the theoretical value of −59 mV/pH for equal numbers of electrons and protons involved in the electrochemical oxidation process. According to previous results [25], the oxidation of epinephrine is a two electrons—two protons electrochemical process. The anodic peak current is almost the same for pH values from 6 to 8, while a decrease in the peak current is observed for pH values of 4 and 5. Consequently, the pH value of 7 was chosen as the optimum value for the analytical applications. This pH value is close to the value characteristic of biological real samples for potential applications in the electroanalysis of neurotransmitters.

**Figure 6:**
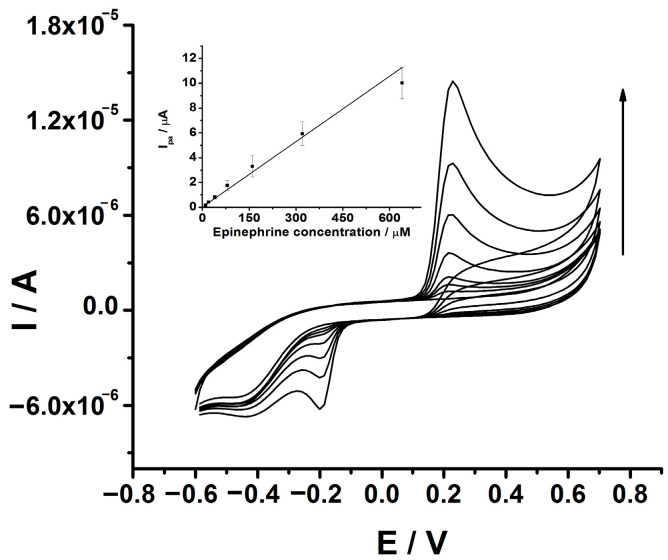
CVs recorded at GCE-PEDOT-AuNPs modified electrode in aqueous solution containing 0.1 M phosphate buffer of pH 7 and various epinephrine amounts of 10, 20, 40, 80, 160, 320, and 640 μM. Potential scan rate: 50 mV/s. Inset: The corresponding anodic peak current versus epinephrine concentration dependence plot. Error bars are incorporated in the calibration plot.

After the optimization of the pH of the solution, the analytical performance of the GCE-PEDOT-AuNPs sensor was estimated in terms of linear response range, sensitivity, limits of detection and quantification, repeatability, and reproducibility. The best analytical response was obtained after a preconcentration step with a duration of 60 s at open circuit potential in the presence of 80 μM epinephrine. In Figure 6, the CV traces recorded at GCE-PEDOT-AuNPs modified electrode in an aqueous buffered solution of pH 7 containing various amounts of epinephrine are depicted. The oxidation of epinephrine takes place at a potential value of 0.24 V vs. Ag/AgCl/KCl (3M). On the cathodic scan, a reduction wave with a peak potential value of (−0.20) V vs. Ag/AgCl/KCl (3M) could be observed. This behavior is characteristic of the catechol moiety of the epinephrine. The anodic peak current increases linearly with the epinephrine concentration in the range of 10 to 640 μM.

The linear regression equation of the anodic peak current versus epinephrine concentration dependence was as follows: *I_pa_* (*μA*) = 1.84 + 0.021 [EPI] (μM), with a correlation coefficient of *r* = 0.9883. The sensitivity of the sensor was estimated from the slope of the calibration plot depicted in the inset of Figure 6. A sensitivity value of 0.021 μA/μM has been obtained. The limits of detection (LD) and quantification (LQ) have been calculated using the equations: *LD* = 3 *Sd*/*m* and *LQ* = 10 *Sd*/*m*, respectively, where *Sd* represents the estimated standard error of the intercept of the regression line, and *m* is the slope of the calibration plot. LD value of 1.4 μM and LQ value of 4.8 μM have been obtained, respectively. The repeatability of the sensor has been estimated by measuring a fixed epinephrine concentration of 80 μM three times with the same sensor and the value was expressed as relative standard deviation (RSD%). A value of 2.1% has been obtained for the estimated repeatability. The reproducibility has been estimated by measuring a fixed epinephrine concentration of 320 μM using three sensors prepared in similar experimental conditions. The RSD% value for reproducibility was 3.4%.

##### Real Sample Application of GCE-PEDOT-AuNPs Sensor for Epinephrine

**Figure 7:**
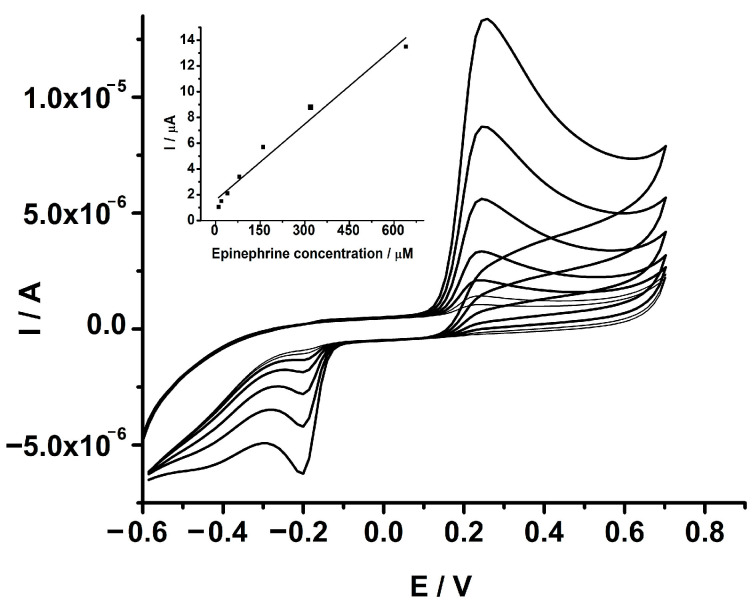
CVs recorded at GCE-PEDOT-AuNPs modified electrode in tap water and 0.04 M PBS, spiked with various epinephrine amounts: 10, 20, 40, 80, 160, 320, and 640 μM. Potential scan rate: 50 mV/s. Inset: The corresponding anodic peak current versus epinephrine concentration dependence plot.

The capability of the GCE-PEDOT-AuNPs sensor has also been investigated by measuring a known epinephrine amount spiked into a sample of tap water at a very low electrolyte concentration of 0.04 M PBS. This application was intended to explore the capability of the sensor to perform analytical measurements in highly resistive media similar to real samples. The presence of epinephrine in wastewater nearby healthcare facilities could be envisaged, and the detection of this target analyte in similar samples has been attempted. The sample was spiked with various amounts of epinephrine from a stock epinephrine solution and the current response was recorded. The CVs recorded at the GCE-PEDOT-AuNPs sensor in tap water spiked with various amounts of epinephrine are depicted in Figure 7.

The oxidation of epinephrine takes place at a potential value of 0.25 V, which is close to the potential value observed in an aqueous buffered solution. There is a linear increase in the anodic peak current due to the oxidation of added epinephrine with the increase in its concentration. The anodic peak current was plotted against the epinephrine concentration and a linear dependence was observed (see inset of Figure 7). The linear regression equation was as follows: *I_pa_* (μA) = 1.57 + 0.020 [*EPI*] (μM), with a correlation coefficient *r* of 0.9874. The slope of the linear regression equation is almost the same as that obtained in an aqueous buffered solution attesting good applicability of the sensor.

Finally, the analytical performance of the proposed sensor is comparable to that of other electrochemical sensors in terms of sensitivity, detection limit, and linear response range (see Table 1). The detection method used in this work is cyclic voltammetry and the obtained figures of merit of the analytical performance are similar to those of electrochemical sensors based on square wave voltammetry and differential pulse voltammetry detection techniques. These findings point out the potential applications of the proposed sensor in the detection of serotonin in similar matrices.

#### 3.2.2. Detection of Serotonin

**Figure 8:**
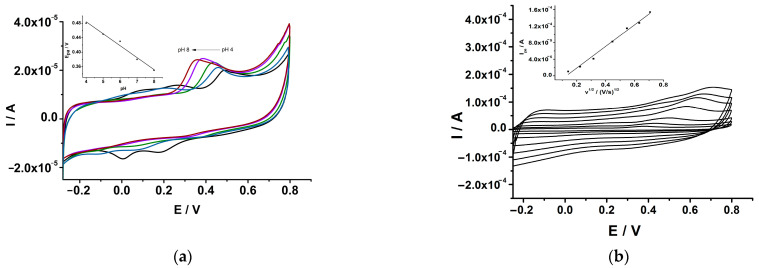
(**a**) CVs recorded at GCE-PEDOT-AuNPs modified electrode in aqueous solution containing 0.1 M phosphate buffer and 320 μM serotonin at pH values of 4 (black), 5 (blue), 6 (green), 7 (magenta), and 8 (red). Potential scan rate: 50 mV/s. Inset: the anodic peak potential versus pH dependence plot. (**b**) CVs recorded at GCE-PEDOT-AuNPs electrode in aqueous solution containing 0.1 M phosphate buffer and 640 μM serotonin at various potential scan rates. Inset: the *I_pa_* versus *v*^1/2^ dependence plot.

The electrochemical detection of serotonin has been investigated at the GCE-PEDOT-AuNPs-based sensor by cyclic voltammetry in an aqueous buffered solution of pH 7. The optimization of the proposed sensor has been performed taking into account the influence of pH on serotonin detection. The influence of pH on the oxidation of serotonin has been studied by cyclic voltammetry in aqueous solutions in the pH range from 4 to 8. The cyclic voltammograms recorded at the GCE-PEDOT-AuNPs sensor in an aqueous solution of various pH values and fixed serotonin concentrations are depicted in Figure 8a. The anodic peak potential *E_pa_* for serotonin oxidation negatively shifts with the increase in pH (see Figure 8a). The linear regression equation was: *E_pa_* (V) = 0.62–0.033 pH, with correlation coefficient *r* = 0.9931. The slope of the *E_pa_* versus pH plot was −33 mV/pH unit attesting to the two electrons and one proton oxidation process [28]. The anodic peak current of serotonin oxidation is the largest for a pH of 7. Therefore, the pH value of 7 has been used in the analytical applications and the estimation of the overall analytical performance of the proposed sensor.

The oxidation of serotonin at the GCE-PEDOT-AuNPs electrode takes place at a potential value of 0.38 V at a pH of 7. The influence of the potential scan rate on the catalytic oxidation of serotonin at the GCE-PEDOT-AuNPs electrode has been investigated (see Figure 8b). There was a linear dependence of the anodic peak current on the square root of the potential scan rate according to the linear regression equation: *I_pa_* (A) = −3.4 × 10^−5^ *+* 2.6 × 10^−4^ *v*^1/2^ (V/s)^1/2^, with the correlation coefficient *r* = 0.9954. This result demonstrates that the oxidation of serotonin at the GCE-PEDOT-AuNPs sensor is a controlled diffusion process. The anodic peak potential was linearly increasing with the logarithm of the potential scan rate according to the equation: *E_pa_* (V) = 0.785 + 0.123 ln*(v)*, correlation coefficient *r =* 0.9989. From the slope of the *Ep* vs. ln*(v)* plot, the number of electrons involved in the oxidation reaction of serotonin has been estimated to be equal to 1 assuming a transfer coefficient α of 0.5.

**Figure 9:**
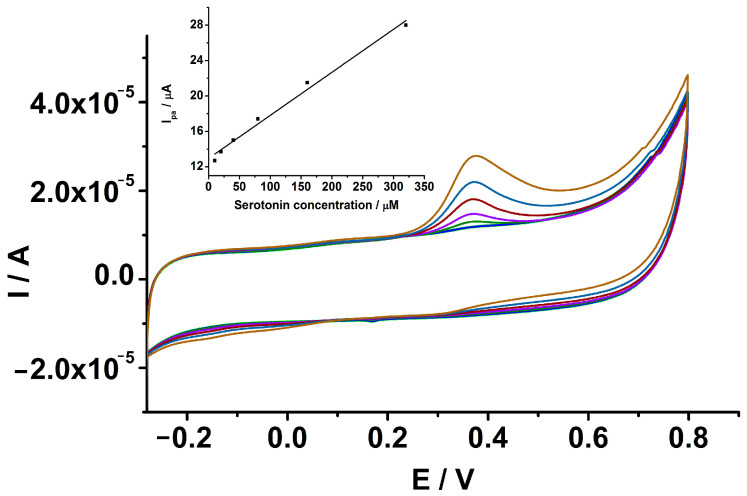
CVs recorded at GCE-PEDOT-AuNPs modified electrode in 0.1 M PBS containing various amounts of serotonin of 10 (dark blue), 20 (green), 40 (magenta), 80 (red), 160 (blue), and 320 (brown) μM. Potential scan rate: 50 mV/s. Inset: The corresponding anodic peak current versus serotonin concentration dependence plot. Error bars are incorporated in the calibration plot.

The analytical response of the GCE-PEDOT-AuNPs-based sensor towards serotonin was investigated. There was a linear increase in the anodic peak current due to the serotonin oxidation with the concentration in the range from 10 to 320 μM (see Figure 9). The linear regression equation was the following: *I_pa_* (μA) *=* 12.9 + 0.049 [*SER*] (μM), with correlation coefficient *r* = 0.9947. From the slope of the calibration plot, the sensitivity of the sensor has been estimated and a value of 0.049 μA/μM has been obtained. The detection and quantification limits have been estimated. A detection limit value of 5.7 μM and a quantification limit value of 18.8 μM serotonin have been obtained, respectively. The repeatability of the proposed sensor has been estimated by measuring three times a serotonin concentration of 320 μM and was expressed as relative standard deviation (RSD%). In this case, a value of 7.2% has been obtained. The reproducibility has been also estimated by measuring the same serotonin concentration with three sensors prepared in similar conditions and a value of 8.9% has been obtained.

**Figure 10:**
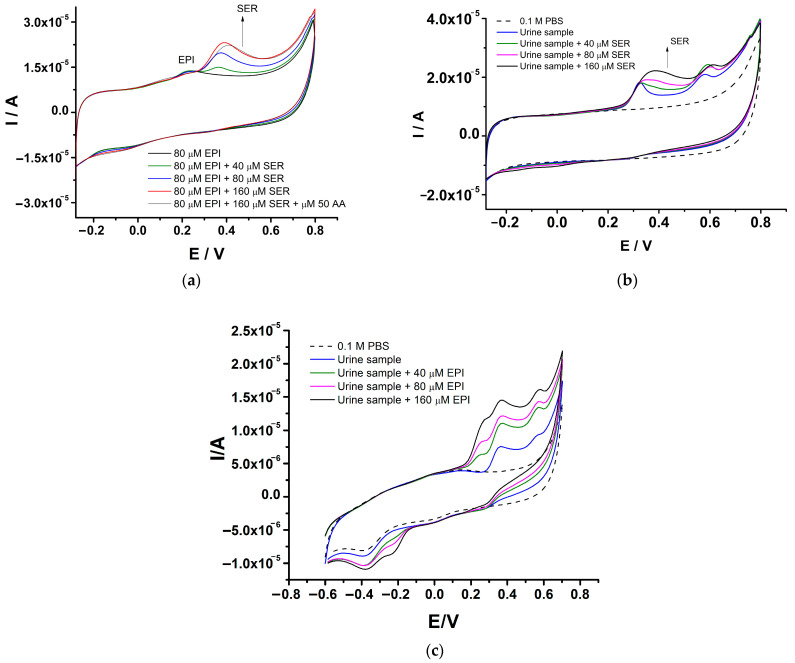
(**a**) CVs recorded at GCE-PEDOT-AuNPs sensor in 0.1 M PBS of pH 7 containing 80 μM epinephrine in the presence of various amounts of serotonin of 40, 80, and 160 μM. Potential scan rate: 50 mV/s. (**b**) CVs recorded at GCE-PEDOT-AuNPs sensor in urine samples spiked with various amounts of 40, 80, and 160 μM serotonin. Potential scan rate: 50 mV/s. (**c**) CVs recorded at GCE-PEDOT-AuNPs sensor in urine samples spiked with various amounts of 40, 80, and 160 μM epinephrine. Potential scan rate: 50 mV/s.

The anti-interference capability of the GCE-PEDOT-AuNPs-based sensor has been investigated by cyclic voltammetry measurements of the epinephrine–serotonin mixture. The selective determination of epinephrine and serotonin in a mixture has been investigated in order to assess the capability of the sensor to measure both species having close oxidation potential values. The study was performed by cyclic voltammetry in an aqueous buffered solution of pH 7 by keeping constant the concentration of epinephrine and increasing the concentration of serotonin. The CVs recorded at the GCE-PEDOT-AuNPs in the epinephrine–serotonin mixture are depicted in Figure 10a.

There is a very good resolution between the peak potentials for epinephrine and serotonin contemporarily present in the mixture. When the serotonin concentration is increased, the corresponding anodic peak current is increasing, while the anodic peak current due to epinephrine oxidation remains unchanged. This behavior demonstrates the possibility to quantify one analyte in the presence of the other without significant interference. The selectivity of the proposed sensor was further investigated by adding in the mixture of the analytes a major interfering specie, that is ascorbic acid (AA). In the presence of AA, the anodic peak current due to epinephrine remains unchanged, while the anodic peak current for serotonin decreases by 4.5% from the initial value and a small positive shift of the serotonin peak potential of 22 mV is observed. These findings point out the capability of the proposed sensor to measure the analytes in a mixture and in the presence of a major interfering specie, a situation that is usually encountered in the electroanalysis of samples with complex composition.

##### Real Sample Application of GCE-PEDOT-AuNPs Sensor for Serotonin

The practical application of the GCE-PEDOT-AuNPs-based sensing platform has been demonstrated by measuring urine samples spiked with known amounts of serotonin (see Figure 10b). The sample was diluted in a ratio 1:10 with 0.1 M phosphate buffer solution and the voltammetric response of the sensor was recorded after each addition. There is an anodic peak located at ca. 0.32 V and a second one situated at ca. 0.58 V. The addition of serotonin resulted in the appearance of an anodic peak potential located at ca. 0.38 V. The corresponding anodic peak current increases with serotonin addition. The recovery values and the relative standard deviation (RSD%) are presented in Table 2. The obtained results demonstrate the good accuracy of the proposed sensing platform for serotonin detection in real samples. In addition, the PEDOT-AuNPs-based sensing platform has been applied in the analysis of urine samples spiked with epinephrine (see Figure 10c). The addition of epinephrine to the urine sample resulted in the appearance of the corresponding anodic peak at ca. 0.24 V specific to epinephrine oxidation. This anodic peak increased linearly with epinephrine concentration. Moreover, there is a good peak potential resolution between the epinephrine oxidation peak and the peaks related to the urine sample. The recovery values including the relative standard deviation for epinephrine are displayed in Table 2. These results confirm the practical applicability of the proposed PEDOT-AuNPs sensing platform in the analysis of real samples.

Subsequently, the comparison of the analytical performance between the proposed GCE-PEDOT-AUNPs sensor and other electrochemical sensors has been performed. Table 3 displays a comparison of the figures of merit of the analytical performance of various electrochemical sensors used in serotonin detection. Compared to other electrochemical sensors, the GCE-PEDOT-AuNPs sensor displayed comparable analytical figures of merit in terms of sensitivity, linear response range, and detection limit. The detection method based on cyclic voltammetry provided comparable analytical performance as in the case of other more sensitive detection techniques like differential pulse voltammetry and square wave voltammetry.

The main drawbacks of the electrochemical preparation procedures like reproducibility, fouling of the electrode surface, and reduced stability over time have been also taken into consideration in the development of the proposed sensing platform. The proposed PEDOT-AuNPs-based sensing platform displayed good reproducibility values for epinephrine and serotonin detection. The obtained reproducibility values expressed as RSD% of 3.4% and 8.9% point out the good analytical performance of the proposed sensor. These values are in the range of those usually reported for electrochemical sensors based on composite-modified electrodes. The anti-fouling capability of the proposed sensing platform has been checked after the analysis of each analyte. The capability of the sensing platform to measure another sample has been checked by measuring the cyclic voltammetric responses in electrolyte solution after the application of the analytical protocol for each analyte. Figure S1 from the Supplementary Material displays the CV traces recorded before and after the application of the analytical protocol for epinephrine detection. The CV traces are well overlapped indicating the very good anti-fouling capability of the PEDOT-AuNPs sensing material. These CVs were recorded after a preconcentration step in the presence of the analyte and the obtained results highlight the good anti-fouling capability of the proposed sensor. A similar result was obtained in the case of serotonin analysis. Figure S2 from Supplementary Material shows the CVs recorded before and after the serotonin analysis and well overlapped voltammograms were obtained. Moreover, the anti-fouling capability of the sensing platform was checked also during several additions of serotonin. In this case, the CVs depicted in Figure S3 clearly demonstrate the good anti-fouling capability of the proposed sensing platform. The stability of the sensing platform was investigated by measuring the analytes at fixed interval times. The anodic peak currents decrease by 9.2% for measuring 160 μM epinephrine (see Figure S4) and by 3.4% for measuring 160 μM serotonin (see Figure S5), respectively.

These results clearly demonstrate the capability of the electrochemical preparation procedure in overcoming some drawbacks associated with the development of electrochemical sensors for biologically active compound analysis.

## 4. Conclusions

The development of a sensing platform based on conducting polymers–noble metal nanoparticles by means of a novel preparation procedure has been achieved. The novel preparation procedure is based on the use of a sinusoidal current superimposed on a constant current. The optimized electrochemical parameters of the SC procedure ensured the successful deposition of the PEDOT-AuNPs sensing material characterized by increased roughness of the polymeric coating and homogenous size distribution and control of the metal nanoparticles. The electrochemical properties of the sensing material have been investigated by cyclic voltammetry and electrochemical impedance spectroscopy techniques and an increased electron transfer capability has been obtained. The sensing platform displayed good overall analytical performance towards the determination of epinephrine with a linear response range over a wide concentration range from 10 to 640 μM, and low detection and quantification limits of 1.4 and 4.8 μM, respectively. In addition, the sensing platform has been successfully applied in the determination of serotonin with good analytical performance in terms of a wide linear response range from 10 to 320 μM serotonin, and detection and quantification limits of 5.7 μM and 18.8 μM serotonin, respectively. The GCE-PEDOT-AuNPs-based sensing platform also displayed good anti-interference capability for measurements of the analytes contemporary present in a mixture and in the presence of an interfering specie like ascorbic acid. The proposed sensing platform has been also successfully applied in the detection of epinephrine and serotonin in real samples with good accuracy and selectivity.

## Figures and Tables

**Table 1:** Comparison of analytical performance of electrochemical sensors for epinephrine.

Electrochemical Sensor	Detection Method	Limit of Detection (M)	Linear Response Range (M)	Sensitivity (µA L mol^−1^)	Sample Matrix	Ref.
NiONP–MWCNT–DHP/GCE	DPV	8.2 × 10^−8^	3.0 × 10^−7^–9.5 × 10^−6^	9.0 × 10^5^	0.2 M PBS (pH = 7)	[9]
Au/SAMs/AuNRs	SWV	6.35 × 10^−6^	1.0 × 10^−5^–6.0 × 10^−5^	9.9 × 10^5^	0.1 M PBS (pH = 7)	[15]
AuNPs/PDA/ANE	DPV	2.6 × 10^−7^	1.0 × 10^−6^–1.0 × 10^−3^	-	0.1 M PBS (pH = 7.4)	[25]
Au-MWCNT-PANI-TiO_2_ Au-MWCNT-PANI-RuO_2_	DPV	1.6 × 10^−7^ 1.8 × 10^−7^	4.9 × 10^−6^–7.69 × 10^−5^	-	0.1 M PBS (pH = 7)	[26]
NP Au film	DPV	2.42 × 10^−6^	2.0 × 10^−5^–1.9 × 10^−4^	-	0.1 M PBS (pH = 7)	[27]
GCE-PEDOT-AuNPs	CV	1.4 × 10^−6^	1.0 × 10^−5^–6.4 × 10^−4^	2.1 × 10^4^	0.1 M PBS (pH = 7)	This work

NiONP—nickel oxide nanoparticles; MWCNT—multilayer carbon nanotubes; DHP—dihexadecylphosphate; GCE—glassy carbon electrode; SAM—self-assembled monolayers; AuNRs—gold nanorods; AuNPs—gold nanoparticles; PDA—polydopamine; ANE—acupuncture needle electrode; AuPt—gold and platinum nanoparticles; GR—graphene; PANI—polyaniline; NP Au—nanoporous gold film; PEDOT—poly(3,4-ethylenedioxythiophene); DPV—differential pulse voltammetry; SWV—square wave voltammetry; CA—chronoamperometry; CV—cyclic voltammetry; PBS—phosphate buffer solution.

**Table 2:** Determination of serotonin and epinephrine in urine samples using the PEDOT-AuNPs-based sensing platform.

Sample	Added (µM)	Detected (µM)	Recovery (%)	RSD (%)
Serotonin				
Sample 1	40	40.6	101.5	5.8
	80	77.6	97.0	8.2
	160	158.8	99.2	5.3
Epinephrine				
Sample 2	40	36.3	90.8	4.8
	80	81.0	101.3	5.9
	160	166.7	104.2	8.4

**Table 3:** Comparison of analytical performance of electrochemical sensors for serotonin.

Electrochemical Sensor	Detection Method	Limit of Detection (M)	Linear Response Range (M)	Sensitivity (µA L mol^−1^)	Sample Matrix	Ref.
Pt/MWCNT/PPy/AgNPs	DPV	1.5 × 10^−7^	5 × 10^−7^–5 × 10^−6^	-	0.2 M PBS (pH = 8)	[11]
PEDOTNTs/rGO/AgNPs/GCE	DPV	1 × 10^−10^	10^−9^–5 × 10^−8^	-	0.1 M PBS (pH = 8)	[12]
AuNPs@PPy/GSPE	SWV	3.32 × 10^−8^	1 × 10^−7^–1.5 × 10^−5^	3.32 × 10^5^	0.02 M PBS (pH = 7.4)	[13]
rGO/PANI/AuNPs@MIPs	DPV	1.17 × 10^−8^	2 × 10^−7^–1 × 10^−5^	-	0.1 M PBS (pH = 7.5)	[16]
GCE-PEDOT-AuNPs	CV	5.7 × 10^−6^	1 × 10^−5^–3.2 × 10^−4^	4.9 × 10^5^	0.1 M PBS (pH = 7)	This work

MWCNT—multiwalled carbon nanotubes; PPy—polypyrrole; AgNPs—silver nanoparticles; AuNPs—gold nanoparticles; PPy—polypyrrole; GSPE—graphite-based screen-printed electrode; rGO—reduced graphene oxide; PANI—polyaniline; MIPs—molecularly imprinted polymers; PEDOTNTs—poly(3,4-ethylenedioxythiophene) nanotubes; GCE—glassy carbon electrode; PEDOT—poly(3,4-ethylenedioxythiophene); SWV—square wave voltammetry; DPV—differential pulse voltammetry; CV—cyclic voltammetry; PBS—phosphate buffer solution.

## Data Availability

The data are contained within the article.

## Supplementary Materials

The following supporting information can be downloaded at: https://www.mdpi.com/article/10.3390/bios13080781/s1, Figure S1: anti-fouling capability of the sensing platform towards epinephrine; Figures S2 and S3: anti-fouling capability of the sensing platform towards serotonin; Figure S4: stability of the sensing platform for epinephrine analysis; Figure S5: stability of the sensing platform for serotonin analysis.
