# Supplementary material for: Electrochemical Sensing Platform Based on Metal Nanoparticles for Epinephrine and Serotonin
Source: Biosensors (Basel). 2023 Aug 1;13(8):781. doi: 10.3390/bios13080781 (PMC10452149; doi:10.3390/bios13080781)
Supplement: Supplementary file 1 [file biosensors-13-00781-s001.zip › biosensors-2508972-supplementary.pdf]

biosensors

Supplementary Materials

Electrochemical Sensing Platform based on Metal Nanoparticles for epinephrine and serotonin

Sorina Alexandra Leau [1,2], Cecilia Lete [1,*], Cristian Matei [3] and Stelian Lupu [2,*]

1 Department of Electrochemistry and Corrosion, Institute of Physical Chemistry "Ilie Murgulescu" of the Romanian Academy, 202 Splaiul Independentei, 060021 Bucharest, Romania
2 Department of Analytical Chemistry and Environmental Engineering, Faculty of Chemical Engineering and Biotechnologies, University "Politehnica" of Bucharest, 1-7 Polizu Gheorghe, 060042 Bucharest, Romania
3 Department of Inorganic, Physical Chemistry and Electrochemistry, Faculty of Chemical Engineering and Biotechnologies, University "Politehnica" of Bucharest, 1-7 Polizu Gheorghe, 060042 Bucharest, Romania
* Correspondence: e-mail@e-mail.com; stelian.lupu@upb.ro (S.L.); clete@icf.ro (C.L.)

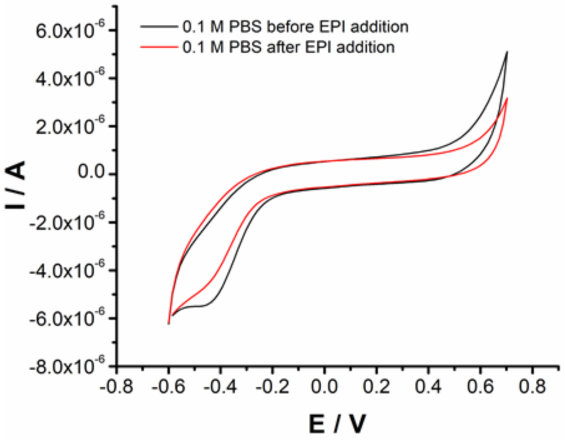

Figure S1. CVs recorded at GCE-PEDOT-AuNPs sensing platform in 0.1 M PBS of pH 7 before and after epinephrine analysis protocol. Potential scan rate: 50 mV/s.

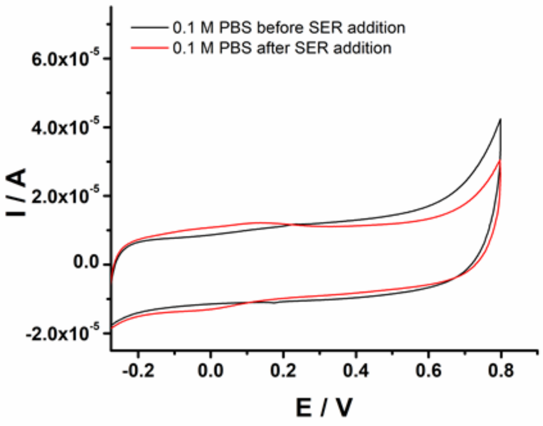

Figure S2. CVs recorded at GCE-PEDOT-AuNPs sensing platform in 0.1 M PBS of pH 7 before and after serotonin analysis protocol. Potential scan rate: 50 mV/s.

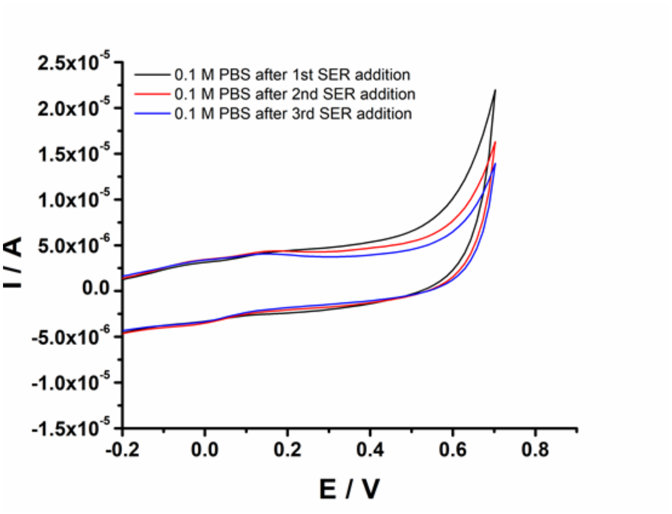

Figure S3. CVs recorded at GCE-PEDOT-AuNPs sensing platform in 0.1 M PBS of pH 7 after 3 consecutive serotonin measurements. Potential scan rate: 50 mV/s.

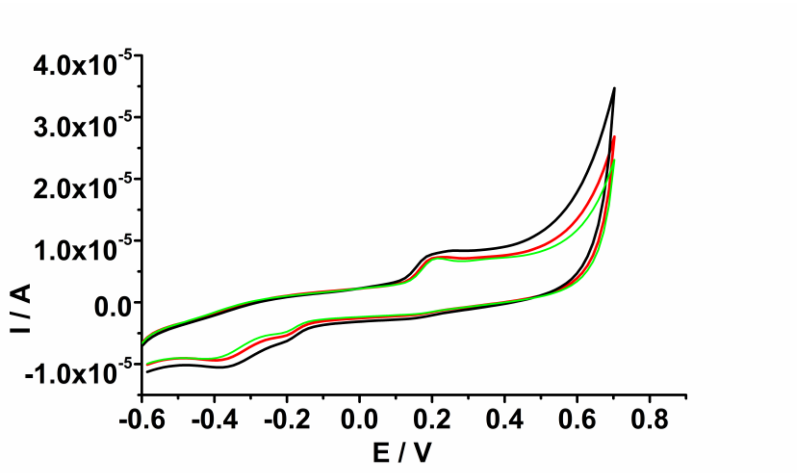

Figure S4. CVs recorded at GCE-PEDOT-AuNPs sensing platform in 0.1 M PBS of pH 7 containing 160 μM epinephrine at different time intervals of 1 (black), 5 (red), and 10 (green) hours. Potential scan rate: 50 mV/s.

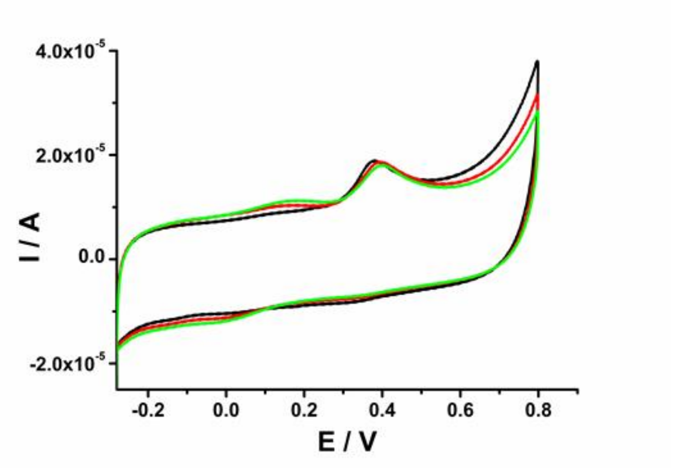

Figure S5. CVs recorded at GCE-PEDOT-AuNPs sensing platform in 0.1 M PBS of pH 7 containing 160 μM serotonin at different time intervals of 1 (black), 5 (red), and 10 (green) hours. Potential scan rate: 50 mV/s.
